# Reproduction of *Meloidogyne enterolobii* on Onion and Potential Yield Suppression

**DOI:** 10.2478/jofnem-2025-0005

**Published:** 2025-04-22

**Authors:** Nabin Poudel, Richard F. Davis, Ted McAvoy, Bhabesh Dutta, Intiaz Amin Chowdhury

**Affiliations:** Department of Plant Pathology, University of Georgia, Tifton, GA 31793; United States Department of Agriculture – Agricultural Research Service, Crop Genetics and Breeding Research Unit, Tifton, GA 31793; Department of Horticulture, University of Georgia, Tifton, GA 31793

**Keywords:** *Allium cepa*, guava root-knot nematodes, nematode reproduction, pathogenicity, red onion, Vidalia onion

## Abstract

*Meloidogyne enterolobii*, is an emerging root-knot nematode species in the southern United States. To date, no studies have evaluated the host status of onions to *M. enterolobii*. This study aimed to assess the reproduction and pathogenicity of *M. enterolobii* on onion cultivars commonly grown in Georgia. Six Vidalia onion cultivars (‘Rio del Sol’, ‘Sapelo’, ‘Sweet Magnolia,’ ‘Tania,’ ‘Vidora,’ and ‘NUN 1011’), three red onion cultivars (‘Red Duke,’ ‘Red Halen,’ and ‘Red Maiden’), and a white onion cultivar (‘Monjablanca’) were evaluated. Each cultivar was inoculated with 8,000 eggs of *M. enterolobii* in a repeated greenhouse trial with six replications each. Twelve weeks post-inoculation, plants were harvested to determine reproduction and pathogenicity based on the reproduction factor (Rf = final nematode population/initial nematode inoculum) and reductions in bulb and shoot weights, respectively. All tested cultivars were susceptible to *M. enterolobii*, with Rf values greater than 1, though significant differences were observed. ‘Vidora’ and ‘Tania’ exhibited the highest galling index and Rf values, while ‘Sweet Magnolia’ and ‘Sapelo’ had the lowest. All red onion cultivars showed significant reductions in weight for both bulbs and shoots, whereas among the Vidalia cultivars, only ‘NUN 1011’ exhibited notable reductions in bulb and shoot weights. These findings suggest that onions are suitable hosts for *M. enterolobii*, and that the nematode's reproduction and pathogenicity vary with onion type and cultivar.

Onion (*Allium cepa*) is one of the economically important vegetable crops grown in the United States. According to the United States Department of Agriculture (USDA) National Agriculture Statistics Service (NASS), the total production of onions in 2023 was approximately 3.32 million tons with an estimated value of $1.5 billion (USDA-NASS, 2024). Georgia is one of the leading onion-producing states in the United States. In 2023, total onion production in Georgia was approximately 115 thousand tons, with an estimated value of $159 million (USDA-NASS, 2024). Georgia is known to produce the Vidalia onion, a short-day type Yellow Granex onion. Vidalia onions are exclusively grown in the southeastern portion of Georgia, where mild winters, low sulfur soils, and sufficient water supply enhance their sweetness (Boyhan et al., 2007). In addition to Vidalia onions, red onions, and white onions are also grown in Georgia.

Onion production in Georgia is constrained by various plant diseases caused by fungi, bacteria, viruses, and plant-parasitic nematodes (Hajihassani et al., 2019; Dutta, 2024). Plant-parasitic nematodes (PPN) are a significant pest in southern Georgia, where relatively warm temperatures create a suitable environment for infection and reproduction on numerous vegetable crops (Marquez et al., 2021). Several PPN genera including cyst nematode (*Heterodora* spp.), lance nematode (*Hoplolaimus* spp.), ring nematode (*Mesocriconema* spp.), root-knot nematodes (*Meloidogyne* spp.), spiral nematode (*Helicotylenchus* spp.), and the stubby-root nematode (*Nanidorus* spp.) have been detected in onion fields of Georgia (Hajihassani et al., 2019; Marquez et al., 2021). Among these, root-knot nematodes (RKN) are one of the state's most prevalent nematode genera infesting onion fields (Hajihassani et al., 2019). Infestation by RKN can cause the formation of small galls in onion roots leading to poor uptake of water and nutrients from the soil. This can lead to stunting, chlorosis, and ultimately a reduction in crop yield (Gregon et al., 2002; Mitkowski et al., 2002).

Multiple species of RKN have been documented to parasitize onions, including *M.arenaria*, *M. chitwoodi*, *M. incognita*, *M. hapla*, *M. javanica*, and *M. graminicola* (Gergon et al., 2002; Mitkowski et al., 2002; Davis and Langston, 2003; Pang et al., 2009a). Moreover, most of these species are capable of causing significant yield loss. For instance, Corgan et al. (1985) observed up to a 76% reduction in onion bulb yield due to *M. incognita* infestation. Gergon et al. (2002) reported yield reductions between 7% and 82% in onion bulb weight under greenhouse conditions when inoculated with 50–10,000 second-stage juveniles (J2) of *M. graminicola* and documented up to a 35% yield loss in field trials. Similarly, total onion weight was reduced by 40.6% to 59.6% in greenhouse conditions when onions were inoculated with *M. hapla* at levels of 3,000 to 12,000 J2/1,500 cm³. In microplot trials inoculated with 160,000 J2 of *M. hapla* per 20,000 cm³ soil, there was a yield reduction of up to 41.3% in onion bulbs (Pang et al., 2009a). These studies clearly show that RKN species can be a major threat in onion production.

In recent years, an aggressive species of RKN (*M. enterolobii* [syn. *M. mayaguensis*]) has been detected in multiple states of the U.S (Philbrick et al., 2020; Hajihassani et al., 2023). This species of RKN is considered one of the most damaging species due to its wide host range, pathogenicity, and ability to develop and reproduce on several crops carrying resistance genes effective against other common RKN species, including *M. incognita*, *M. javanica*, and *M. arenaria* (Castagnone-Sereno, 2012; Philbrick et al., 2020). In Georgia, *M. enterolobii* was first detected in 2021 from a sweet potato field in Tattnall County (Hajihassani et al., 2023), where approximately 40% of the onions in the state are produced (Stubbs, 2024). Sweet potatoes, often rotated with onions in this region of Georgia, are excellent hosts for *M. enterolobii*. This rotation enables the *M. enterolobii* galled root debris and second-stage juveniles to remain in the soil after sweet potato cultivation, increasing the likelihood of onions encountering the nematode. There is limited information about the interactions of *M. enterolobii* with onions. To our knowledge, no study has shown whether *M. enterolobii* can parasitize onions. Hence, this study aimed to assess the reproductive ability and pathogenicity of *M. enterolobii* on different onion cultivars grown in Georgia. The findings from this study are critical for developing effective management strategies to mitigate the economic losses caused by this nematode.

## Materials and Methods

### Plant materials

Six Vidalia onion cultivars (‘Sapelo,’ ‘Sweet Magnolia,’ ‘Tania,’ ‘Vidora,’ ‘Rio del Sol,’ and ‘NUN 1011’), three red onion cultivars (‘Red Halen,’ ‘Red Duke,’ and ‘Red Maiden’), and one white onion cultivar (‘Monjablanca’) were evaluated in this study. All the selected cultivars are commercially grown in Georgia. Nine-weeks-old transplants of all onion cultivars were sourced from the University of Georgia, Vidalia Onion and Vegetable Research Center in Lyons, GA.

### Preparation of nematode inoculum

The *M. enterolobii* isolate used in this trial was originally isolated from sweet potatoes in South Georgia (Hajihassani et al., 2023) and was used as the inoculum source. A pure culture of *M. enterolobii* was maintained on eggplant cv. ‘Black Beauty’ and tomato cv. ‘Rutger’ for three months before use. To prepare the inoculum, eggs and second-stage juveniles were extracted by soaking galled roots in a 0.5% sodium hypochlorite solution while rotating at 250 rpm for two minutes. After shaking, roots and sodium hypochlorite solution were poured through mesh sieves (150 μm, 75 μm, and 25 μm), rinsed with tap water, and collected in a 50 ml tube. The rinsate collected from a sieve was then subjected to the centrifugal sugar flotation to separate eggs from other debris. Specifically, the rinsate was centrifuged at 3000 rpm for 5 minutes. The supernatant was discarded, and the pellet was resuspended in a sucrose solution (454 g/L). This suspension was then centrifuged at 3000 rpm for 1 minute. The egg supernatant was poured over a 25 μm sieve and rinsed thoroughly with tap water to remove the sugar (Jenkins, 1964). Eggs were collected from the sieve and stored in tap water at 4°C for 24 hours before inoculation. After extracting the eggs, the hatching percentage of nematode eggs was calculated. For the hatching experiment, a Kimwipe paper was placed over a metal basket, which was positioned above a bowl filled with water until the paper just touched the water's surface. The egg suspension containing 8,000 eggs was then poured onto the Kimwipe paper, and the entire hatching setup was placed in an incubator at a temperature of 25 ± 1°C. Emerging J2s were collected daily over a 25 μm mesh sieve for seven days. The hatching percentage was calculated as the total number of J2s recovered divided by the initial number of eggs poured over the hatching setup.

### Establishment of the trial

Trials were conducted in greenhouse conditions at the University of Georgia, Tifton Campus. The first trial was conducted during spring 2023 (January– March), and the second trial was conducted during spring 2024 (January–March). Plastic pots with 20 cm diameter were filled with sterilized sandy loam soil. Each pot was planted with a single 9-weeks-old onion seedling with two to four true leaves. A week after planting the onion seedlings, each pot was inoculated with approximately 8,000 eggs of *M. enterolobii*. Eggs were sourced exclusively from eggplant in the first experiment and tomato in the second to ensure that the inoculum remained consistent within each trial. The suspension of eggs was inoculated into three 1.5-cm-deep holes made 1 cm from the base of the onion seedlings, and then the holes were covered with a light layer of soil and moistened with water. Treatments included *M. enterolobii*-inoculated and non-inoculated control pots for each onion cultivar, and the pots were arranged in a randomized complete block design with six replications. Inoculated pots allowed the evaluation of nematode reproduction, and the comparison of inoculated and non-inoculated pots allowed the evaluation of the nematode's effect on plant growth. Plants were watered daily, and each pot was fertilized with 3 g of Osmocote 15-9-12 N-P-K controlled-release fertilizer (Scotts-Sierra Horticultural Products Co, Marysville, OH, U.S.A.) at the time of planting and 2 g after one and a half month of planting.

### Data collection

The trials were terminated 12 weeks after inoculation. At harvest, plant materials, including shoots, roots, and bulbs, were gently separated from the soil and wrapped in a paper towel. Each plant's fresh shoot, root, and bulb weight were then recorded on a weighing scale. Roots were then examined to determine the root gall index. Galling indices were based on a rank scale described by Marquez and Hajihassani (2023), which was adapted from Taylor and Sasser (1978) and Bridge and Page (1980), where 0 = no galls and 1 = 1 to 2, 2 = 3 to 10, 3 = 11 to 30, 4 = 31 to 100 galls. Plants with more than 100 root galls were rated as 5 = 25%, 6 = 50%, and 7 = 75% of roots are galled; 8 = roots are completely galled; 9 = roots are completely galled and rotting; and 10 = dead plant. After rating the galling indices, eggs were extracted using the sodium hypochlorite-based extraction method described previously. However, 1% sodium hypochlorite solution was used for at-harvest egg extraction for increased extraction efficiency, unlike inoculum preparation where 0.5% sodium hypochlorite was used to ensure the viability of eggs (Hussey and Barker, 1973; Marquez and Hajihassani, 2023). Eggs were enumerated with a nematode counting slide (Chalex LLC, Park City, UT, U.S.A) under an inverted compound microscope (ZEISS Axio Vert.A1, Jena, Germany) at 40X magnification. The reproduction factor (Rf) was calculated as the number of eggs extracted at harvest divided by the initial inoculum level adjusted for the percentage hatch. Initial inoculum was 3,600 for the first trial (45 % hatch of 8,000 eggs) and 3,120 for the second trial (39 % hatch of 8,000 eggs). These inoculum levels represent typical RKN density (1-2 J2/cm^3^ soil) observed in vegetable fields of Georgia. Roots and shoots of each onion seedlings were dried at 80°C for 120 hours to measure dry weights.

### Data analysis

Data were analyzed using R studio version 2021.09.0 Build 351 (R Core Team, 2020). Cultivars and trials were considered as a fixed effect and replications as a random effect in a linear mixed model, and data from two trials were pooled where no cultivars × trials effect existed at *P* = 0.05. In a scenario where the cultivars × trials effect existed, data for each trial were analyzed separately. Data for the number of eggs per root system, number of eggs per gram of root, Rf, and galling index were log^10^ (x) transformed to meet the assumptions of normality. However, non-transformed means of replicates from each trial are presented in the table. Mean comparisons were performed using Tukey's Honestly Significant Difference (HSD) at α = 0.05. To compare the mean difference in growth and yield between inoculated and non-inoculated onion plants of each variety, two sample-independent *t*-tests were performed at α = 0.05. Before performing the *t*-test, assumptions for the *t*-test, including normality of the dependent variable and homogeneity of variances among the independent samples, were tested.

## Results

### Nematode population, root gall index, and reproduction Factor

There was a significant trial x cultivar interaction for the number of eggs per root system, number of eggs per gram of root, and the Rf. Therefore, data were analyzed separately for each trial. However, there was no trial x cultivar interaction for root gall index, so data from both trials were pooled for this parameter.

Significant differences were observed among the tested onion cultivars for the number of eggs per root system in both trials (Table 1). In the first trial, ‘Vidora’ supported the most eggs per root system, significantly greater than the counts for ‘Sapelo’ and ‘Sweet Magnolia’ but statistically like the other onion cultivars tested. The number of eggs per root system for ‘Tania,’ ‘NUN 1011’, ‘Monjablanca,’ ‘Rio del Sol,’ ‘Red Duke,’ ‘Red Halen,’ and ‘Red Maiden’ were intermediate and did not differ from any cultivar tested. In the second trial, ‘Tania’ had the most eggs per root system, significantly greater than those recorded for ‘Sapelo,’ ‘Sweet Magnolia,’ ‘Monjablanca,’ and ‘Rio del Sol’ (Table 1). The number of eggs per root system for ‘Vidora,’ ‘NUN 1011’, ‘Red Duke,’ ‘Red Halen,’ and ‘Red Maiden’ were intermediate and did not differ from any cultivar tested.

**Table 1:** Susceptibility of ten onion cultivars to *Meloidogyne enterolobii*.

**Onion cultivars**	**Number of eggs/roots system**		**Number of eggs/grams of root**		**Reproduction factor (Rf)^x^**	
		
	**Trial 1**	**Trial 2**	**Trial 1**	**Trial 2**	**Trial 1**	**Trial 2**
Monjablanca	17,190^y^ ab^z^	10,560 b	2,223 ab	1,447 b	4.78 ab	3.38 b
Rio del Sol	16,280 ab	12,978 b	1,982 ab	1,596 b	4.52 ab	4.16 b
Sapelo	11,546 b	13,667 b	1,407 b	1,709 b	3.21 b	4.58 b
Sweet Magnolia	11,997 b	11,574 b	1,474 b	1,441 b	3.33 b	3.71 b
Tania	22,999 ab	28,160 a	2,806 ab	3,657 a	6.39 ab	9.03 a
Vidora	27,930 a	20,915 ab	3,406 a	2,477 ab	7.76 a	6.70 ab
NUN 1011	21,137 ab	20,519 ab	2,632 ab	2,374 ab	5.87 ab	6.58 ab
Red Duke	20,322 ab	20,415 ab	2,257 ab	2,258 ab	5.64 ab	6.54 ab
Red Halen	21,373 ab	17,883 ab	2,466 ab	2,042 ab	5.94 ab	5.73 ab
Red Maiden	17,947 ab	16,090 ab	2,145 ab	1,886 b	4.99 ab	5.16 ab

***P*-value**	0.01	0.0012	0.0251	0.0042	0.01	0.0012

x Reproduction factor, Rf = final total egg count/initial inoculum egg count.

y Values are the mean of six replicates per trial.

z Values followed by the same letter within a column are not significantly different according to Tukey's HSD test (α = 0.05).

Significant differences were observed among onion cultivars for the number of eggs per gram of root in both trials (Table 1). In the first trial, ‘Vidora’ had the most eggs per gram of root and was significantly greater than ‘Sapelo’ and ‘Sweet Magnolia,’ but not significantly different from the other cultivars tested. The number of eggs per gram of root for ‘Monjablanca,’ ‘Tania,’ ‘NUN 1011,’ ‘Red Duke,’ ‘Red Halen,’ and ‘Red Maiden’ were intermediate and statistically similar to all other cultivars tested. In the second trial, ‘Tania’ had the most eggs per gram of root, which was significantly greater than ‘Sweet Magnolia,’ ‘Monjablanca,’ ‘Sapelo,’ ‘Rio del Sol,’ and ‘Red Maiden.’ The number of eggs per gram of root in ‘Vidora,’ ‘NUN 1011,’ ‘Red Duke,’ and ‘Red Halen’ were intermediate and did not differ from any cultivar tested.

The Rf varied significantly among onion cultivars in both trials. In the first trial, ‘Vidora’ had the highest Rf, which was significantly greater than those observed for ‘Sapelo’ and ‘Sweet Magnolia’, but not significantly different from the other cultivars tested (Table 1). The Rf values for ‘Tania,’ ‘NUN 1011’, ‘Red Duke,’ ‘Red Halen,’ ‘Monjablanca,’ ‘Rio del Sol,’ and ‘Red Maiden’ were intermediate and did not differ from any cultivar tested. In the second trial, ‘Tania’ had the highest Rf, significantly higher than ‘Monjablanca,’ ‘Rio del Sol,’ ‘Sapelo,’ and ‘Sweet Magnolia’ (Table 1). The Rf values for ‘Vidora,’ ‘NUN 1011’, ‘Red Duke,’ ‘Red Halen,’ and ‘Red Maiden’ were intermediate and did not differ from any cultivar tested.

**Figure 1:**
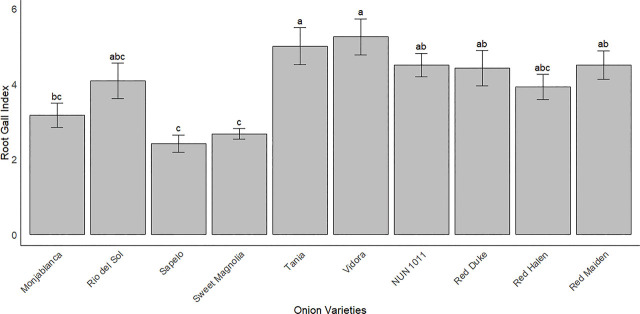
Root gall indices for cultivars of onion following *M. enterolobii* inoculation (0 to 10 scale). Bars represent the Root Gall Index (mean ± standard error). Values are pooled data from two trials. Bars with the same letter are not significantly different according to Tukey's HSD test (α = 0.05).

There were significant differences among the onion cultivars for galling index (Fig. 1). ‘Tania’ and ‘Vidora’ had the highest galling index, which was significantly greater than ‘Sapelo,’ ‘Sweet Magnolia,’ and ‘Monjablanca,’ but similar to other cultivars tested.

### Pathogenicity of *M. enterolobii* on onion

**Figure 2:**
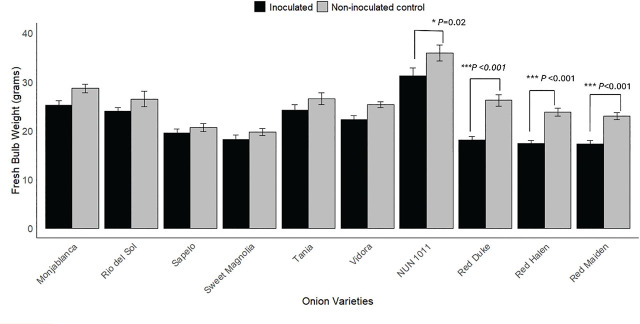
Effect of *Meloidogyne enterolobii* on fresh bulb weights of onion cultivars. Bars represent the fresh bulb weight (mean ± standard error). Values are pooled data from two trials. Two independent *t*-tests were done in two samples to identify differences between inoculated and non-inoculated control treatments. ^*^ and ^***^ represent the 5% and 0.1% level of significance, respectively.

*Meloidogyne enterolobii* caused a significant reduction in bulb weights of all three red onion cultivars tested (Fig. 2). For ‘Red Duke,’ ‘Red Halen,’ and ‘Red Maiden,’ the average fresh bulb weight without *M. enterolobii* inoculation was statistically greater than the bulb weight obtained from the inoculated plants. Nematode parasitism reduced the bulb weight of ‘Red Duke’ by 31.1%, ‘Red Halen’ by 26.6%, and ‘Red Maiden’ by 24.6%. Among the Vidalia onion cultivars, only ‘NUN 1011’ showed a significant reduction in bulb weight (17.4%). The other Vidalia onion cultivars tested had numerically lower fresh bulb weights following *M. enterolobii* inoculation, but the differences were not statistically different. A similar result was obtained for Monjablanca, where the differences in bulb weight between inoculated and non-inoculated control treatments were not statistically different.

**Figure 3:**
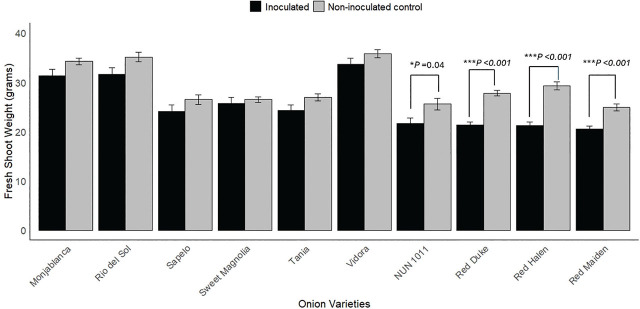
Effect of *Meloidogyne enterolobii* on fresh shoot weights of onion cultivars. Bars represent the fresh shoot weight (mean ± standard error). Values are pooled data from two trials. Two sample-independent *t*-tests were done to identify differences between inoculated and non-inoculated control treatments. ^*^ and ^***^ represent the 5% and 0.1% level of significance, respectively.

**Figure 4:**
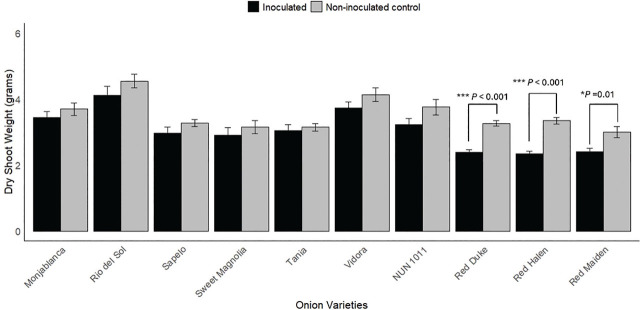
Effect of *Meloidogyne enterolobii* on dry shoot weights of onion. Bars represent the fresh bulb weight (mean ± standard error). Values are pooled data from two trials. Two sample independent *t*-tests were done to identify differences between inoculated and non-inoculated control treatments. ^*^ and ^***^ represent the 5% and 0.1% level of significance, respectively.

There were significant differences in the fresh shoot weights of all three red onion cultivars between inoculated and non-inoculated control treatments (Fig. 3). Nematode parasitism reduced the fresh shoot weight of ‘Red Duke’ by 23.2%, ‘Red Halen’ by 27.4%, and ‘Red Maiden’ by 22.2%. A similar trend was observed for dry shoot weight; all three red onion cultivars significantly reduced dry shoot weight when inoculated with *M. enterolobii* (Fig. 4). Among Vidalia onion cultivars, only ‘NUN 1011’ showed a significant reduction in fresh shoot weight, with a reduction of 22.2%; the dry shoot weight of ‘NUN 1011’ was not significantly affected by nematode parasitism. There was no statistical difference in fresh and dry shoot weight of Monjablanca between inoculated and non-inoculated treatments. There was a clear trend in which all cultivars tested had numerically lower fresh and dry shoot weights following *M. enterolobii* inoculation. Still, the only statistically significant differences were those noted above (Fig. 3, 4). There were also no statistical differences among the fresh or dry root weights of the onion cultivars tested (data not shown).

## Discussion

*Meloidogyne enterolobii* is emerging as a significant threat to the vegetable industry in the United States. This species of RKN has been documented in multiple states, including Florida, Georgia, Louisiana, North Carolina, and South Carolina. *Meloidogyne enterolobii* affects a variety of weeds and crops, including vegetables, fruit trees, and ornamental plants (Brito et al., 2007; Philbrick et al., 2020; Hajihassani et al., 2023; Schwarz et al., 2024). In Georgia, *M. enterolobii* was first detected in 2021 from a sweet potato field in Tattnall County (Hajihassani et al., 2023), located at the heart of Georgia's world-famous Vidalia onion-producing region. Importantly, most of the sweet potato cultivars that have intermediate to high levels of resistance to the southern RKN, *M. incognita,* are susceptible to *M. enterolobii* (Galo et al., 2024), which increases the risk of damage to onion when the onion is rotated with sweet potato in fields infested by *M. enterolobii*. Moreover, the sandy soils and warm temperatures of Georgia's onion-growing region foster conditions conducive to *Meloidogyne enterolobii* infestation in onions (Koenning et al., 1996; Marquez et al., 2021). Compared to *M. incognita*, *M. enterolobii* has a relatively shorter life cycle and thus can have rapid population build-up and increased host damage (Collett et al., 2024). Yet, the literature has no information regarding the onion's hosting ability to *M. enterolobii*. To more accurately assess the potential for damage and the need to manage *M. enterolobii* in onion, it is necessary to evaluate the host status of onion to *M. enterolobii*.

Our study showed that Vidalia, red, and white onions are good hosts to *M. enterolobii,* with Rf values ranging from 3.2 to 9.0. Though all the cultivars tested showed good hosting ability to *M. enterolobii*, significant differences were observed among the cultivars. ‘Vidora’ and ‘Tania’ had significantly higher Rf values compared to other cultivars, which documents that there can be significant differences among onion cultivars in *M. enterolobii* reproduction. This result is reminiscent of the results obtained by Kotcon et al. (1985), where the authors reported significant differences among onion cultivars for reproducing *M. hapla*. Likewise, in a screening assay done by Pang et al. (2009b), there was a significant difference among onion cultivars to the reproduction of *M. hapla*, with some cultivars showing resistance. However, in our study, none of the tested cultivars of either Vidalia or red onions showed resistance to *M. enterolobii*. ‘Sapelo’ and ‘Sweet Magnolia’ recorded the lowest number of eggs per root system and eggs per gram root in both experiments and statistically lower than ‘Tania’ and ‘Vidora’ in one of the two experiments, showing that there can be differences in onion cultivars for susceptibility to *M. enterolobii*.

Our study showed differences in plant growth between the non-inoculated control and the inoculated treatment for some cultivars by the end of the trial. All three red onion cultivars significantly reduced growth and yield parameters like fresh shoot weight, fresh bulb weight, and dry shoot weight. In contrast, only one Vidalia onion cultivar (‘NUN 1011’) showed a reduction in these parameters. The percentage reduction of these growth parameters was significantly higher in red onion cultivars than in Vidalia onion cultivars and white onion cultivars, which suggests that the pathogenicity of *M. enterolobii* can be different among onion types. Although the eggs per gram of fresh root weight in red onion cultivars were either statistically lower or comparable to those in Vidalia onion cultivars and a white onion cultivar, the percentage reduction in bulb weight and shoot weight was still significantly higher in the red onion cultivars.

In our study, *M. enterolobii* had no significant effect on the growth parameters of a white onion cultivar and most Vidalia onion cultivars, except for ‘NUN 1011.’ However, a general trend of numerical reduction in plant weight was observed in inoculated white and Vidalia onion cultivars, affirming the potential of *M. enterolobii* to damage the onion crops (Fig. 2, 3, and 4). *Meloidogyne enterolobii* might need higher inoculum densities than we used in our trial to reduce the yield of Vidalia onion cultivars. The hatching rate of *M. enterolobii* in our trials was 45% in the first trial and 39% in the second, resulting in an inoculation density of 3,600 J2/1,500 cm³ soil in the first trial and 3,120 J2/1,500 cm³ soil in the second. A study by Pang et al. (2009a) found that while *M. hapla* began to reduce onion growth and yield at an inoculation density of 3,000 J2/1,500 cm³ soil, maximum yield reduction occurred at 10,000 J2/1,500 cm³ soil. Similarly, Gergon et al. (2002) observed a yield reduction ranging from 7% to 82% in onions inoculated with 50 to 10,000 second-stage juveniles (J2) of *M. graminicola*. Further investigation into the correlation between *M. enterolobii* inoculum density and yield reduction in Vidalia onion cultivars is necessary to determine whether higher densities could lead to more significant yield losses. It is also important to note that the response of onions to RKN may differ between greenhouse and field conditions. For example, onions inoculated with *M. hapla* showed significant yield reductions in both environments. Still, there was a greater reduction in total onion weight in greenhouse conditions compared to microplot trials, despite using the same inoculum density (Pang et al., 2009a). Gergon (2002) also observed variations in the pathogenicity of *M. graminicola* between greenhouse and field trials, with greater yield reductions in greenhouse conditions. Therefore, conducting trials in *M. enterolobii*-infested fields would provide valuable insights into the pathogenicity of *M. enterolobii* in large-scale onion production.

Previous studies have shown different species of RKN, including *M. incognita*, *M. chitwoodi*, *M. graminicola*, and *M. hapla,* significantly reduce the yield of onion. However, before this work, the response of onion to *M. enterolobii* was not known. To the best of our knowledge, this is the first data to begin evaluating the reaction of onion cultivars to *M. enterolobii*. In summary, this study identified Vidalia onion, white onion, and red onion as a host to *M. enterolobii* and suggested differences in the response of onion types and cultivars to *M. enterolobii* reproduction and pathogenicity.
